# Genetic Variation in the *REL* Gene Increases Risk of Behcet’s Disease in a Chinese Han Population but That of *PRKCQ* Does Not

**DOI:** 10.1371/journal.pone.0147350

**Published:** 2016-01-19

**Authors:** Feilan Chen, Lei Xu, Tingting Zhao, Xiang Xiao, Yongquan Pan, Shengping Hou

**Affiliations:** 1 Chongqing Medical University, Chongqing, P. R. China; 2 Chongqing Engineering Research Center For Rodent Laboratory Animals, Chongqing, P. R. China; 3 The First Affiliated Hospital of Chongqing Medical University, Chongqing, P. R. China; 4 Chongqing Key Laboratory of Ophthalmology, Chongqing, P. R. China; University of Birmingham, UNITED KINGDOM

## Abstract

Genome-wide association studies (GWAS) and candidate gene studies have identified the *REL* and *PRKCQ* genes as risk loci for various autoimmune diseases. The purpose of the present study was to investigate the association of the *REL* and *PRKCQ* genes with Behcet’s disease (BD) in a Chinese Han population. A case-control study was conducted on three single nucleotide polymorphisms (SNPs), rs13031237, rs702873, and rs842647 of the *REL* gene and three SNPs (rs4750316, rs11258747, and rs947474) of the *PRKCQ* gene using polymerase chain reaction-restriction fragment length polymorphism (PCR-RFLP) in a total of 623 BD patients and 1,074 healthy controls. Multiple variables were assessed, including age, sex distribution, and extra-ocular findings. In the present study, the frequencies of rs842647 GG genotypes and rs842647 G alleles were significantly higher in patients than in controls and those of the rs842647 AG genotypes were lower in patients than in controls [GG genotype: Bonferroni corrected *P*-value for gender adjustment (*P*_c_^a^) = 0.0074, odds ratio (OR) = 1.63; G allele: *P*_c_^a^ = 0.0072, OR = 1.57; AG genotype: *P*_c_^a^ = 0.024, OR = 0.63, respectively]. No statistically significant differences in the frequencies of rs702873, rs13031237, rs4750316, rs11258747, and rs947474 between BD patients and controls were observed. Stratification analysis indicated that the *REL* rs842647 polymorphism was associated with BD patients with skin lesions. No significant association of the other five SNPs between BD patients with other extra-ocular findings, including genital ulcer, arthritis, and positive pathergy test results was found. The *REL* rs842647 polymorphism may be a susceptibility factor for BD pathogenesis and skin lesions, which indicate that c-Rel may be involved in the pathogenesis and skin lesions of BD through the NF-κB pathway.

## Introduction

Behcet’s disease (BD) is a refractory multisystem immune-mediated disease with recurrent episodes of uveitis, multiform skin lesions, oral aphthae, and genital ulceration [1]. Although the etiology of BD remains poorly defined, it is generally believed, as for many autoimmune or auto-inflammatory disorders, that genetic variants triggered by certain environmental factors contribute to its development. Genome-wide association studies (GWAS) and candidate gene studies have identified the predominant BD susceptibility loci within the *major histocompatibility complex (MHC) class I* region, which include *human leukocyte antigen-B51* (*HLA-B51*) and *HLA-A26* [2–5]. However, these genes only partially explain the genetic risk of BD, suggesting that other *non-MHC* genes remain to be discovered. Recently, GWAS established an association between the *IL10*, *IL23R-IL12RB2*, *STAT4*, and *GIMAP* genes and BD [3, 4, 6]. Candidate gene association studies have involved searches for additional BD genes, including the *GIMAP* gene in European patients, the *NFKB1* and *NFKBIA* genes in Turkish patients, and the *CD40* gene in the Chinese patients [7–9]. However, the genes that have been associated to BD do not fully explain its pathogenesis, and no study focusing on the identification of a novel BD susceptibility loci has been conducted.

The nuclear factor-κB (NF-κB) signal transduction pathway plays a crucial role in T-cell development in autoimmunity disorders and inflammation diseases. The *REL* gene is located on chromosome 2 and encodes for c-Rel, which is a member of the NF-κB family. c-Rel is expressed solely in mature hematopoietic cells and plays a critical role in T-cell development, antigen-presenting cell function, and the CD40 signaling pathway by the formation of pro-inflammatory cytokines and regulating the expression of genes. Previous studies have shown that *Rel* knockout mice do not develop autoimmune diseases [10, 11]. In addition, the *PRKCQ* and *CD40* genes are involved in the c-Rel signal pathway, thus suggesting that these genes share a common disease pathway. Based on the important roles that *REL* and *PRKCQ* genes play in the pathology of autoimmune diseases, the association between polymorphisms in the *REL* and *PRKCQ* genes and BD was investigated. Here, three *REL* SNPs (rs13031237, rs702873, and rs842647) and three *PRKCQ* SNPs (rs4750316, rs11258747, and rs947474) were selected as candidate risk variants of BD. The present study detected higher frequencies of the rs842647 GG genotype and rs842647 G allele in BD patients. Stratified analysis also showed an association between the rs842647 G allele polymorphism and skin lesions in BD patients.

## Materials and Methods

### Ethics Statement

Prior to enrolling in the present study, all subjects, including BD patients and controls, provided their written, informed consent. In the case of the pediatric BD patients, their parents provided written and informed consent. All procedures were in according to the tenets of the Declaration of Helsinki. The Clinical Research Ethics Committee of the First Affiliated Hospital of Chongqing Medical University and Ethics Committee of Chongqing Medical University approved the study (Permit Number: 2009–201008).

### Study Population

A total of 623 BD patients with ocular involvement and 1,074 age- and geographic-area-matched normal controls with no history of any ocular or autoimmune disease were enrolled in the present study, and all patients and controls were of Han Chinese ethnicity. All blood samples were obtained from the First Affiliated Hospital of Chongqing Medical University (Chongqing, China) and Zhongshan Ophthalmic Center, Sun Yat-sen University (Guangzhou, China) from April 2005 to December 2013. BD was diagnosed according to the criteria of the International Study Group for BD [12]. The clinical characteristics of BD patients are summarized in Table 1.

**Table 1:** Clinical characteristics of the BD patients.

Extraocular findings	n (Total = 623)	%
Age at onset (years **±** SD)	33.73 ± 8.93	
Males	540	86.68
Females	83	13.32
Uveitis	623	100
Oral ulcer	623	100
Genital ulcer	372	59.7
Skin lesions	481	77.2
Arthritis	98	15.7
Positive pathergy test results	113	18.1

### Genomic DNA Extraction

Peripheral blood samples were gathered from all study participants by venipuncture. Genomic DNA extraction was obtained from peripheral blood of controls and patients using the QIAmp DNA Blood Mini Kit (QIAGEN Inc., Hilden, Germany) and was reserved at -80°C until analysis.

### Genotyping and Quality Control

*REL* polymorphisms (rs13031237, rs702873, and rs842647) and *PRKCQ* polymorphisms (rs4750316, rs11258747, and rs947474) were genotyped by polymerase chain reaction-restriction fragment length polymorphism (PCR-RFLP). Amplification of the target DNA fragment of *REL* and *PRKCQ* genes was conducted by PCR using the primers designed using Primer Premer5.0 and restriction enzymes presented in Table 2. Each PCR reaction was conducted in a 10-μl reaction mixture containing 5 μL of a Go Taq^®^ Green Master Mix (Promega Corporation, Madison, WI, USA), 20 pmol of each primer, and 0.2 μg of genomic DNA. The conditions were as follows: initial denaturation at 95°C for 5 min, followed by 35 cycles of denaturation at 95°C for 30 s, annealing at different temperatures (62°C for rs13031237, rs842647, rs702873, and rs4750316, 60°C for rs947474 and rs11258747) for 40 s, extension at 72°C for 30 s, and a final extension at 72°C for 3 min. PCR products of the rs13031237, rs842647, rs702873, rs947474, rs11258747, and rs4750316 polymorphisms were digested with 2 U of *Csp6I* (Thermo Fisher Scientific Inc., Ontario, Canada), *HpyCH4 III* (Thermo Fisher Scientific Inc., Ontario, Canada), BsiWI (New England BioLabs, Inc., Ontario, Canada), *DdeI* (Thermo Fisher Scientific Inc., Ontario, Canada), *Mwol* (New England BioLabs, Inc., Ontario, Canada) and *HpyCH4 III* (Thermo Fisher Scientific Inc., Ontario, Canada) restriction enzymes (Table 2) in a 10-μL reaction mixture for 12–16 h. Digestion products were separated via electrophoresis on a 4.5% agarose gel and stained with GoldView (SBS Genentech Beijing, China). Genotypes were evaluated in a masked fashion, and analysis of all ambiguous samples was repeated. Moreover, 10% of the samples were double-checked to validate the results of the PCR-RFLP using direct sequencing (Sangon Biotech, Co., Ltd., Shanghai, China).

**Table 2:** Primers and restriction enzymes used for RFLP analysis of the *REL* and *PRKCQ* genes.

Gene	rs number	Primers	Restriction enzyme
*REL*	rs842647	5′-TGCTTGTCTCTGATTCTCTGGGTC-3′ 5′-CTGGGCGACAAGTGTGAAACTC-3′	*HpyCH4III*
	rs13031237	5'-GAGTTGTTATGAGAGTAAAAGGCTGC-3' 5'-AAGTACACAAGTTCTGCCTAGGGTAA-3'	*Csp6I*
	rs702873	5'-CAAAGCATCCTTCTTACTGGGTGT-3' 5'-AAGGCATTAGGAAGATTAGTGGTGTC-3'	*BsiWI*
*PRKCQ*	rs947474	5'-ACCAGTTATGAAGGGTGACAAAGA-3' 5'- GATCAAATACCAACTGCGTTGACT-3'	*DdeI*
	rs4750316	5'-GGAAGAGCTGATAAGGGAAATGTC-3' 5'-TCCAGAAGGGCCAGAACACTA-3'	*HpyCH4III*
	rs11258747	5'-GGGTCAATCTCCTTCCGTTCA-3' 5'-TGCTCTGCTCCCTTTCAGCTCTT -3'	*MwoI*

RFLP: restriction fragment length polymorphism

### Statistical Analysis

Statistical analysis was conducted using SPSS version 17.0 for Windows (SPSS Inc., Chicago, IL, U.S.). Hardy-Weinberg equilibrium (HWE) was conducted by using the χ^2^ test. Genotype and allele frequencies were evaluated by direct counting and were compared between patients and controls using the χ^2^ test or Fisher’s exact test. Logistic regression analysis was performed to assess the influence of gender on the association of the polymorphisms to this disease and to assess the association of the tested SNPs to the extra-ocular findings. All statistical tests were two-sided, and statistical significance was set at *P* < 0.05.

## Results

No statistically significant differences in age distribution between BD patients [average age: 33.73 (8.93) years; 540 males and 83 females] and controls [average age 39.61 (8.61) years; 570 males and 504 females] were observed. However, the BD patient group contained about six times more male participants than female participants (Table 1). The clinical features of BD patients are shown in Table 1.

The distribution of genotype frequencies of the six tested SNPs in the control group conformed to HWE (*P* > 0.05). The genotype and allele frequencies of the three tested *REL* polymorphisms and *PRKCQ* polymorphisms in the patients and controls are shown in Table 3. In the present study, BD patients showed a higher frequency of the rs842647 GG genotype (83.63%) than that observed in healthy controls (75.79%) [Bonferroni corrected *P* value (*P*_c_) = 0.0036, OR = 1.63]. However, BD patients showed a lower frequency for the rs842647 AG genotype (15.25%) than the controls (22.25%) (*P*_c_ = 0.011, OR = 0.63). The G allele frequency of the rs842647 was statistically significantly higher in BD patients (91.25%) than in healthy controls (86.92%) (*P*_c_ = 0.0034, OR = 1.57). To evaluate the influence of gender on the association of rs842647 to the *REL* gene, we conducted a binary logistic regression analysis and found that differences remained significant after a binary logistic regression analysis (GG genotype: *P*_c_^a^ = 0.0074, AG genotype: *P*_c_^a^ = 0.024, G allele: *P*_c_^a^ = 0.0072, respectively). No differences in the frequencies of rs702873, rs13031237, rs4750316, or rs947474 between BD patients and controls were observed.

**Table 3:** Frequencies of alleles and genotypes of *REL* and *PRKCQ* polymorphisms in patients and controls.

SNP	GenotypeAllele	Cases (N = 623)	Controls (N = 1,074)	*P-*value	*P*_c_	*P-*value^a^	*P*_*c*_^*a*^	OR (95% CI)
rs842647	AA	7 (1.12)	21 (1.96)	0.195	NS	0.23	NS	0.57 (0.24–1.35)
	AG	95 (15.25)	239 (22.25)	0.00047	0.011	0.001	0.024	0.63 (0.48–0.82)
	GG	521 (83.63)	814 (75.79)	0.00015	0.0036	0.00031	0.0074	1.63 (1.27–2.10)
	A	109 (8.75)	281 (13.08)	0.00014	0.0034	0.000302	0.0072	0.64 (0.50–0.80)
	G	1,137 (91.25)	1,867 (86.92)	0.00014	0.0034	0.000302	0.0072	1.57 (1.24–1.98)
rs702873	AA	14 (2.25)	29 (2.70)	0.567	NS	NS	NS	0.83 (0.43–1.58)
	AG	136 (21.83)	257 (23.93)	0.323	NS	NS	NS	0.89 (0.70–1.12)
	GG	473 (75.92)	788 (73.37)	0.246	NS	NS	NS	1.14 (0.91–1.44)
	A	164 (13.16)	315 (14.66)	0.226	NS	NS	NS	0.88 (0.72–1.08)
	G	1,082 (86.84)	1,833 (85.34)	0.226	NS	NS	NS	1.13 (0.93–1.39)
rs13031237	GG	600 (96.31)	1,032 (96.09)	0.821	NS	NS	NS	1.06 (0.63–1.78)
	GT	23 (3.69)	42 (3.91)	0.82	NS	NS	NS	0.94 (0.56–1.58)
	G	1,223 (98.15)	2,106 (98.04)	0.82	NS	NS	NS	1.06 (0.64–1.77)
	T	23 (1.85)	42 (1.96)	0.82	NS	NS	NS	0.94 (0.56–1.58)
rs4750316	CC	510 (81.86)	893 (83.15)	0.50	NS	NS	NS	0.92 (0.71–1.19)
	GC	104 (16.69)	174 (16.20)	0.79	NS	NS	NS	1.04 (0.80–1.35)
	GG	9 (1.44)	7 (0.65)	0.103	NS	NS	NS	2.23 (0.83–6.03)
	C	1,124 (90.21)	1,960 (91.25)	0.31	NS	NS	NS	0.88 (0.70–1.12)
	G	122 (9.79)	188 (8.75)	0.31	NS	NS	NS	1.13 (0.89–1.44)
rs11258747	GG	550 (88.28)	928 (86.41)	0.27	NS	NS	NS	1.19 (0.88–1.60)
	GT	72 (11.56)	144 (13.41)	0.27	NS	NS	NS	0.84 (0.62–1.14)
	TT	1 (0.16)	2 (0.19)	0.90	NS	NS	NS	0.86 (0.08–9.52)
	G	1,172 (94.06)	2,000 (93.11)	0.28	NS	NS	NS	1.17 (0.88–1.56)
	T	74 (5.94)	148 (6.89)	0.28	NS	NS	NS	0.85 (0.64–1.14)
rs947474	AA	422 (67.74)	736 (68.53)	0.74	NS	NS	NS	0.96 (0.78–1.19)
	AG	174 (27.93)	311 (28.96)	0.65	NS	NS	NS	0.95 (0.76–1.18)
	GG	27 (4.33)	27 (2.51)	0.04	NS	NS	NS	1.76 (1.02–3.02)
	A	1,018 (81.70)	1,783 (83.01)	0.33	NS	NS	NS	0.91 (0.76–1.10)
	G	228 (18.30)	365 (16.99)	0.33	NS	NS	NS	1.09 (0.91–1.31)

*P*_c_: Bonferroni corrected *P*-value

*P*_c_^a^: Bonferroni-corrected *P*-value for gender adjustment

^a^: Gender-adjusted *P*-value

CI: confidence interval; OR: odds ratio

The associations of the 6 SNPs with clinical characteristics of BD, including genital ulcer, skin lesions, arthritis, and positive pathergy tests were then investigated. The frequencies of GG genotype and G allele of rs842647/REL were significantly higher in patients with skin lesions than in controls (GG genotype: *P*_c_^a^ = 0.029, G allele: *P*_c_^a^ = 0.034, respectively) (Table 4, S1 Table). No significant association of the other five SNPs was detected between BD patients with those clinical findings and controls.

**Table 4:** Frequencies of alleles and genotypes of rs842467/*REL* polymorphisms in patients with skin lesions and controls.

SNP	GenotypeAllele	Cases (N = 481)	Controls (N = 1074)	*P-*value	*P*_c_	*P-*value^a^	*P*_c_^a^	OR (95% CI)
rs842647	AA	6 (1.25)	21 (1.96)	0.32	NS	0.37	NS	0.63 (0.25–1.58)
	AG	73 (15.18)	239 (22.25)	0.0013	0.031	0.0022	NS	0.63 (0.47–0.83)
	GG	402 (83.57)	814 (75.79)	0.00059	0.014	0.0012	0.029	1.63 (1.23–2.15)
	A	85 (8.84)	281 (13.08)	0.00068	0.016	0.0014	0.034	0.64 (0.50–0.83)
	G	877 (91.16)	1,867 (86.92)	0.00068	0.016	0.0014	0.034	1.55 (1.20–2.01)

*P*_c_: Bonferroni corrected *P*-value

*P*_c_^a^: Bonferroni-corrected *P*-value for gender adjustment

^a^: Gender-adjusted *P*-value

CI: confidence interval; OR: odds ratio

## Discussion

The present study investigated the association of *REL* polymorphisms (rs13031237, rs702873, and rs842647) and *PRKCQ* polymorphisms (rs4750316, rs11258747, and rs947474) with BD in a Han Chinese population. The results indicated that the frequencies of the GG genotype and G allele of rs842647 were positively associated with BD patients in this Han Chinese population. No association with BD for the two tested SNPs polymorphisms (rs702873, rs842647) in the *REL* gene or the three tested SNPs polymorphisms (rs4750316, rs11258747, and rs947474) in *PRKCQ* gene in the Han Chinese population was detected.

In the present study, the *REL* and *PRCKQ* genes were selected as candidates for the recently reported association of the *REL* and *PRCKQ* gene with other autoimmune diseases.

The three SNPs in the *REL* gene that were tested in the present study, namely, rs13031237, rs702873, and rs842647, have been associated with RA [13], SLE [14], and celiac disease [15]. Because BD is considered as an immune-mediated disease and thus may share similar genetic risk factors, rs13031237, rs702873, and rs842647 of *REL* were selected as candidate SNPs in the present study. The SNPs rs4750316, rs11258747, and rs947474 in the *PRKCQ* gene, which encodes a protein kinase C isoform involved in NF-κB activation and T-helper cell subset differentiation, were selected as SNP candidates based on their associations with RA, T1DM, and psoriasis. The following efforts were made for quality control. First, non-Han individuals were excluded to avoid interference from differences in genetic ancestry, and the genotype distribution of controls and patients conformed to the HWE. In addition, the diagnostic criteria of patients were performed in strict accordance with previously described basis. Then, 10% of the samples were randomly chosen for sequencing to validate the genotype results using PCR-RFLP, and the results were determined to be absolutely in agreement with those of the first genotyping results.

The intronic rs842647 GG polymorphism in the *REL* gene has been previously reported as a risk factor for potential CD in Southern Italy populations, and the expression of the c-REL gene was higher in Marsh 0 potentials CD patients than in controls [16]. Similar to the findings of previous studies, a positive association between the GG genotype and G allele of rs842647 with BD was detected, which suggests that the rs842647 GG genotype is a common predisposing factor for BD and potential CD. The SNP rs13031237 T allele has been identified as a risk factor for RA in *UK* cohorts and SLE in Chinese population [13, 17]. The rs702873 G allele was earlier identified as risk factor for psoriasis in populations in the U.K. and Ireland [18]. No association between intronic SNP rs13031237 and rs702873 SNP at *REL* and susceptibility to BD was detected in the Han Chinese population. In the case of the other three tested SNPs, rs4750316, rs11258747, and rs947474 in the *PRKCQ* gene, no association with BD patients in the Han Chinese population was detected. These results were not consistent with those of previous studies, including those covering the association of *PRKCQ* rs4750316 with RA in European populations and that of rs947474 and rs11258747 with T1D in British cohorts [19–21]. These difference may be because BD, which is known as an immune-related disease, may have distinct underlying pathogenic mechanisms that are influenced by distinct genetic loci compared with RA and T1D.

The relationship between the clinical characteristics of patients with *REL* and *PRKCQ* polymorphisms was also assessed. Those clinical characteristics included genital ulcers, skin lesions, arthritis, and positive pathergy tests. The rs842647 GG genotype and G allele was associated with an increased risk for skin lesions in BD patients. These findings indicate that the *REL* rs842647 polymorphism is associated not only with the occurrence of disease but also with the clinical characteristics of disease. No relationship between any of the other five tested SNP polymorphisms or clinical characteristics of patients was observed.

BD is generally regarded as a T cell-mediated disease attributed by a Th1, Th17, and Th22 immune responses and their respective cytokines such as IFN-γ, TNF-α, IL-17, IL22, and IL-23 [22]. NF-κB has been indicated to play a crucial role in the pathogenesis of BD via the regulation of apoptosis-related factors, as well as contribute to the resistance of T cells to apoptosis [23]. Moreover, c-Rel, a unique member of the vertebrate NF-κB family, has recently been shown to be required for the development of Treg cells, Th1 cells, and Th17 cells in EAE [10, 24], as well as for the production of IL23 subunit by the dendritic cells. In addition, the *PRKCQ* gene, which encodes a protein kinase C isoform, a key regulator of TCR-mediated NF-κB activation pathway, is associated with RA and T1D susceptibility. The current study showed that the *REL* gene was associated with BD susceptibility, whereas the *PRKCQ* was not, suggesting that the NF-κB signaling pathway gene, *REL*, and not *PRKCQ* plays an essential role in BD pathogenesis.

The current study has a few limitations. The results have identified the association between *REL* and *PRKCQ* polymorphisms with subjects from a Han Chinese population. Future replication studies that include other ethnic groups should therefore be performed. Furthermore, the BD patients in the present study were predominantly male. Sex-matched samples are thus needed to confirm the results in BD. In addition, it is not yet known whether the observed *REL* rs842647 polymorphism has a biological function in REL in relation to BD pathogenesis, as well as contributes to BD pathogenesis. The fact that NF-κB signaling pathway has a role in the pathogenesis of BD may provide new ways of managing this eye disease.

In conclusion, the results of the current study indicate that *REL* rs842647 may influence susceptibility to BD and that REL may be involved in the BD pathogenesis and clinical features. No association between any of the other tested SNPs with BD in the Han Chinese population was detected.

## Supporting Information

S1 Table Supporting Table. (DOC)

## References

[1] Sakane T, Takeno M, Suzuki N, Inaba G. Behcet's disease. The New England journal of medicine. 1999;341(17):1284–91. 10.1056/NEJM199910213411707 . 10528040

[2] Ohno S, Ohguchi M, Hirose S, Matsuda H, Wakisaka A, Aizawa M. Close association of HLA-Bw51 with Behcet's disease. Arch Ophthalmol. 1982;100(9):1455–8. . 6956266 10.1001/archopht.1982.01030040433013

[3] Meguro A, Inoko H, Ota M, Katsuyama Y, Oka A, Okada E, et al Genetics of Behcet disease inside and outside the MHC. Ann Rheum Dis. 2010;69(4):747–54. 10.1136/ard.2009.108571 . 19684014

[4] Mizuki N, Meguro A, Ota M, Ohno S, Shiota T, Kawagoe T, et al Genome-wide association studies identify IL23R-IL12RB2 and IL10 as Behcet's disease susceptibility loci. Nat Genet. 2010;42(8):703–6. 10.1038/ng.624 . 20622879

[5] Remmers EF, Cosan F, Kirino Y, Ombrello MJ, Abaci N, Satorius C, et al Genome-wide association study identifies variants in the MHC class I, IL10, and IL23R-IL12RB2 regions associated with Behcet's disease. Nat Genet. 2010;42(8):698–702. 10.1038/ng.625 20622878 PMC2923807

[6] Lee YJ, Horie Y, Wallace GR, Choi YS, Park JA, Choi JY, et al Genome-wide association study identifies GIMAP as a novel susceptibility locus for Behcet's disease. Ann Rheum Dis. 2013;72(9):1510–6. 10.1136/annrheumdis-2011-200288 . 23041938

[7] Yenmis G, Oner T, Cam C, Koc A, Kucuk OS, Yakicier MC, et al Association of NFKB1 and NFKBIA polymorphisms in relation to susceptibility of Behcet's disease. Scandinavian journal of immunology. 2015;81(1):81–6. 10.1111/sji.12251 . 25367031

[8] Ortiz-Fernandez L, Conde-Jaldon M, Garcia-Lozano JR, Montes-Cano MA, Ortego-Centeno N, Castillo-Palma MJ, et al GIMAP and Behcet disease: no association in the European population. Annals of the rheumatic diseases. 2014;73(7):1433–4. 10.1136/annrheumdis-2013-205156 . 24625627

[9] Hou S, Yang Z, Du L, Jiang Z, Shu Q, Chen Y, et al Identification of a susceptibility locus in STAT4 for Behcet's disease in Han Chinese in a genome-wide association study. Arthritis Rheum. 2012;64(12):4104–13. 10.1002/art.37708 . 23001997

[10] Chen G, Hardy K, Pagler E, Ma L, Lee S, Gerondakis S, et al The NF-kappaB transcription factor c-Rel is required for Th17 effector cell development in experimental autoimmune encephalomyelitis. Journal of immunology. 2011;187(9):4483–91. 10.4049/jimmunol.1101757 . 21940679

[11] Lamhamedi-Cherradi SE, Zheng S, Hilliard BA, Xu L, Sun J, Alsheadat S, et al Transcriptional regulation of type I diabetes by NF-kappa B. J Immunol. 2003;171(9):4886–92. . 14568969 10.4049/jimmunol.171.9.4886

[12] Weichsler B, Davatchi F, Mizushima Y, Hamza M, Dilsen N, et al Criteria for diagnosis of Behcet's disease. International Study Group for Behcet's Disease. Lancet. 1990;335(8697):1078–80. . 1970380

[13] Gregersen PK, Amos CI, Lee AT, Lu Y, Remmers EF, Kastner DL, et al REL, encoding a member of the NF-kappaB family of transcription factors, is a newly defined risk locus for rheumatoid arthritis. Nature genetics. 2009;41(7):820–3. 10.1038/ng.395 19503088 PMC2705058

[14] Varade J, Palomino-Morales R, Ortego-Centeno N, Diaz-Rubio M, Fernandez-Gutierrez B, Gonzalez-Gay MA, et al Analysis of the REL polymorphism rs13031237 in autoimmune diseases. Ann Rheum Dis. 2011;70(4):711–2. 10.1136/ard.2010.134593 . 20876593

[15] Trynka G, Zhernakova A, Romanos J, Franke L, Hunt KA, Turner G, et al Coeliac disease-associated risk variants in TNFAIP3 and REL implicate altered NF-kappaB signalling. Gut. 2009;58(8):1078–83. 10.1136/gut.2008.169052 . 19240061

[16] Sperandeo MP, Tosco A, Izzo V, Tucci F, Troncone R, Auricchio R, et al Potential celiac patients: a model of celiac disease pathogenesis. PloS one. 2011;6(7):e21281 10.1371/journal.pone.0021281 21760890 PMC3132737

[17] Zhou XJ, Lu XL, Nath SK, Lv JC, Zhu SN, Yang HZ, et al Gene-gene interaction of BLK, TNFSF4, TRAF1, TNFAIP3, and REL in systemic lupus erythematosus. Arthritis Rheum. 2012;64(1):222–31. 10.1002/art.33318 21905002 PMC3994469

[18] Genetic Analysis of Psoriasis C, the Wellcome Trust Case Control C, Strange A, Capon F, Spencer CC, Knight J, et al A genome-wide association study identifies new psoriasis susceptibility loci and an interaction between HLA-C and ERAP1. Nature genetics. 2010;42(11):985–90. 10.1038/ng.694 20953190 PMC3749730

[19] Raychaudhuri S, Remmers EF, Lee AT, Hackett R, Guiducci C, Burtt NP, et al Common variants at CD40 and other loci confer risk of rheumatoid arthritis. Nat Genet. 2008;40(10):1216–23. 10.1038/ng.233 18794853 PMC2757650

[20] Cooper JD, Smyth DJ, Smiles AM, Plagnol V, Walker NM, Allen JE, et al Meta-analysis of genome-wide association study data identifies additional type 1 diabetes risk loci. Nat Genet. 2008;40(12):1399–401. 10.1038/ng.249 18978792 PMC2635556

[21] Barrett JC, Clayton DG, Concannon P, Akolkar B, Cooper JD, Erlich HA, et al Genome-wide association study and meta-analysis find that over 40 loci affect risk of type 1 diabetes. Nat Genet. 2009;41(6):703–7. 10.1038/ng.381 19430480 PMC2889014

[22] Park UC, Kim TW, Yu HG. Immunopathogenesis of ocular Behcet's disease. Journal of immunology research. 2014;2014:653539 10.1155/2014/653539 25061613 PMC4100451

[23] Todaro M, Zerilli M, Triolo G, Iovino F, Patti M, Accardo-Palumbo A, et al NF-kappaB protects Behcet's disease T cells against CD95-induced apoptosis up-regulating antiapoptotic proteins. Arthritis Rheum. 2005;52(7):2179–91. 10.1002/art.21145 . 15986355

[24] Hilliard BA, Mason N, Xu L, Sun J, Lamhamedi-Cherradi SE, Liou HC, et al Critical roles of c-Rel in autoimmune inflammation and helper T cell differentiation. The Journal of clinical investigation. 2002;110(6):843–50. 10.1172/JCI15254 12235116 PMC151124

