# Supplementary material for: Genetic Variation in the REL Gene Increases Risk of Behcet’s Disease in a Chinese Han Population but That of PRKCQ Does Not
Source: PLoS One. 2016 Jan 19;11(1):e0147350. doi: 10.1371/journal.pone.0147350 (PMC4718718; doi:10.1371/journal.pone.0147350)
Supplement: S1 Table — (DOC) [file pone.0147350.s001.doc]

**S1 Table Frequencies of alleles and genotypes of rs842467/*REL* polymorphisms in patients with skin lesions and controls**

| **SNP** | **Genotype**  **Allele** | **Cases**  **(N =481)** | **Controls**  **(N =1074)** | ***P-*value** | ***P*_c_** | ***P-*value^a^** | **P_c_^a^** | **OR**  **(95% CI)** |
| --- | --- | --- | --- | --- | --- | --- | --- | --- |
| rs842647 | AA | 6 (1.25) | 21 (1.96) | 0.32 | NS | 0.37 | NS | 0.63 (0.25–1.58) |
|  | AG | 73 (15.18) | 239 (22.25) | 0.0013 | 0.031 | 0.0022 | NS | 0.63 (0.47–0.83) |
|  | GG | 402 (83.57) | 814 (75.79) | 0.00059 | 0.014 | 0.0012 | 0.029 | 1.63 (1.23–2.15) |
|  | A | 85 (8.84) | 281 (13.08) | 0.00068 | 0.016 | 0.0014 | 0.034 | 0.64 (0.50–0.83) |
|  | G | 877 (91.16) | 1,867 (86.92) | 0.00068 | 0.016 | 0.0014 | 0.034 | 1.55 (1.20–2.01) |

*P*_c_ : Bonferroni corrected *P*-value

*P*_c_^a^: Bonferroni-corrected *P*-value for gender adjustment

^a^: Gender-adjusted *P*-value

CI: confidence interval; OR: odds ratio
